# Clove-Chitosan Nanoparticles Alleviate Fertility Impairment and Reproductive Dysfunction in Obese Male Rat Model

**DOI:** 10.1007/s43032-026-02076-w

**Published:** 2026-04-01

**Authors:** Sama Yassen Ahmed, Ayman Saber Mohamed, Ahmed Imam Dakrory, Mennatallah H. Abdelaziz

**Affiliations:** Zoology Department, Faculty of Science, Cairo University, Giza, Egypt

**Keywords:** Clove, Chitosan, Nanoparticles, High fat diet, Male infertility, Oxidative stress

## Abstract

**Background:**

Male infertility, often linked to obesity, is associated with hormonal imbalance, oxidative stress, and poor sperm quality. Recent studies suggest that treatment with clove and chitosan offers promising results by reducing oxidative damage, improving sperm parameters, and restoring testicular function, making this combination a potential therapeutic approach for enhancing male fertility in obese individuals. On this, the current study was conducted to assess the protective impacts and the promising role of the chitosan nanoparticles (ChNPs) and the clove-chitosan nanoparticle (CNPs) against infertility caused by the high-fat diet (HFD) in rats.

**Methods:**

CNPs were produced and characterized using UV–Visible spectroscopy, X-ray diffraction, and transmission electron microscopy. Hyperlipidemia causes male infertility by induction of HFD for 4 weeks. 24 rats were divided into four groups (n = 6) as follows: Control: were fed on regular chow pellets. HFD: were fed on HFD. HFD + ChNPs: were fed on HFD along with ChNPs (45 mg/kg.b.wt). HFD + CNPs: were fed on HFD along with CNPs (45 mg/kg.b.wt).

**Results:**

compared to the HFD model group, administration of ChNPs and CNPs revealed a significant decline in final body weight and weight gain and a significant increase in sex hormone levels, total proteins, HDL-cholesterol, sperm motility, sperm count, seminal fructose level, glutathione reduced, and catalase, along with optimizing testicular architecture. Furthermore, CNPs caused a significant decrease in glucose, cholesterol, LDL-cholesterol, triglycerides, sperm abnormality, malondialdehyde, and nitric oxide. CNPs treatment helps repair and restore the germinal epithelium and shows normal seminiferous tubules and have the leading effect than ChNPs.

**Conclusion:**

CNPs alleviate testicular dysfunction caused by HFD by reducing hyperlipidemia and hyperglycemia, restoring sex hormone levels, enhancing spermatogenesis, and balancing oxidants-antioxidants system. CNPs showed a synergistic effect on fertility compared to chitosan and clove used independently.

## Introduction

Infertility is a major health problem and has been found to occur in approximately 15% of couples. In modern society, the incidence of male infertility has increased because of several factors, including environmental pollution, stress, and lifestyle. Male infertility has social and psychological impacts on everyday life [1]. A high-fat diet (HFD), defined by an increased intake of dietary fats relative to carbohydrates and proteins, is associated with weight gain, insulin resistance, and chronic low-grade inflammation, which play a crucial role in developing obesity and its related comorbidities [2]. A HFD contributes to male infertility through hormonal dysregulation and increased oxidative stress. Excessive adipose tissue leads to elevated aromatase activity, which converts testosterone into estradiol, reducing serum testosterone levels and disrupting the hypothalamic-pituitary–gonadal axis. This hormonal imbalance negatively affects spermatogenesis, reducing sperm concentration, motility, and morphology [3].

In addition to hormonal and oxidative effects, obesity induces physical changes that impair fertility. For instance, increased scrotal fat elevates testicular temperature, negatively affecting sperm production and quality [4]. Recent decades have witnessed a considerable fall in sperm quality among humans, which has been directly connected to reproductive problems [5]. This trend highlights the confusing nature of obesity as a condition that leads to numerous health complications, including reproductive dysfunctions [6]. In addition, obesity causes hormonal imbalances and disrupts the hypothalamic–pituitary–gonadal (HPG) axis, leading to reduced testosterone levels and elevated estrogen levels. This hormonal imbalance can impair spermatogenesis, lower sperm quality, and diminish libido, ultimately affecting male fertility [7]. Furthermore, the testes, being highly susceptible to oxidative damage, exhibit significant reductions in antioxidant defense mechanisms under high-fat diet conditions. Increased lipid peroxidation in testicular tissue, a key marker of oxidative stress, has been strongly correlated with diminished sperm quality, reduced motility, and overall impaired male fertility [8].

Nanoparticles (NPs) have emerged as promising tools in treating male infertility due to their unique physicochemical properties, including their nanoscale size, large surface area-to-volume ratio, and capacity for surface modification. These features enable NPs to deliver therapeutic agents, such as antioxidants, hormones, or genes, directly to the male reproductive system, enhancing efficacy while minimizing systemic side effects [9]. For instance, oxidative stress is a major contributing factor to male infertility, often resulting in sperm DNA damage and reduced motility. Antioxidant-loaded nanoparticles have the potential to scavenge reactive oxygen species (ROS) within the testes, thereby improving sperm quality and function [10]. In addition to delivering antioxidants, nanoparticles can also serve as carriers for hormones like follicle-stimulating hormone (FSH) or testosterone, allowing for sustained and targeted release, which can help regulate spermatogenesis in hypogonadal or idiopathic infertile males [11].

Chitosan is typically produced under alkaline conditions or via enzymatic treatment. Its chemical structure, degree of deacetylation, and molecular weight are crucial in determining its physical and chemical properties [12]. Chitosan is highly valued for its biodegradability, biocompatibility, antimicrobial properties, and ability to form films and gels. These unique characteristics make it a versatile material with applications across various industries such as biomedicine, agriculture, food, and water treatment [13]. The relationship between chitosan and obesity has been studied extensively, with evidence supporting its potential role in weight management through various mechanisms. Studies indicate that chitosan helps regulate cholesterol and triglyceride levels, improving overall lipid metabolism [14].

Clove (*Syzygium aromaticum*), the dried flower buds of the clove tree, are widely recognized for their culinary, medicinal, and cultural significance. Native to the Maluku Islands of Indonesia, they have been used for centuries as spices, natural preservatives, and traditional remedies [15]. The relationship between clove and obesity has been examined in various studies, focusing on its active compounds, particularly eugenol, and their metabolic and anti-obesity properties. Eugenol, the primary aromatic compound in clove oil, has shown potential in reducing adipogenesis (fat cell formation) and enhancing metabolic health [16]. Studies suggest that eugenol can inhibit lipid accumulation in fat cells and promote the breakdown of stored fat, potentially aiding obesity management [17]. Dietary eugenol has also been found to mitigate the effects of a high-fat diet, including reducing skeletal muscle atrophy and fat accumulation, indicating its potential therapeutic benefits for managing obesity-related complications [18].

Additionally, clove's rich antioxidant profile may contribute to its anti-obesity effects by reducing oxidative stress and inflammation, which are closely linked to obesity and metabolic disorders [19]. Moreover, Clove has been shown to reduce testicular damage caused by hypoxia (oxygen deprivation). Its antioxidant activity helps alleviate oxidative stress in the testes, leading to improved sperm parameters and the restoration of normal testicular function [20]. Clove's antioxidant compounds protect sperm cells from oxidative stress-related damage, contributing to enhanced sperm viability and count, essential for male fertility [21]. Interestingly, a clove-chitosan nanoparticle (CNPs) is better than using either compound by itself because it is more stable, releases drugs more slowly, and works better because it combines the biocompatibility and mucoadhesive qualities of chitosan with the active compounds from clove. The nanoparticles protect the compounds in cloves, which helps them target better and stay at the site of action longer, which makes their antibacterial and antioxidant benefits stronger [22].

On this, the current study was conducted to assess the protective impacts and the promising role of the clove nanoparticles against infertility caused by the high-fat diet in rats after 4 weeks of follow-up.

## Materials and Methods

### Chemicals and Reagents

Chitosan (MW is 750,000, degree of deacetylation is 80%) and sodium alginate were purchased from Sigma-Aldrich (St. Louis, MO, USA), Clove was purchased from the local market in Cairo city. Luteinizing hormone (LH), follicular stimulating hormone (FSH), and testosterone hormone Elissa kits (DRG International Co. USA). Fructose, reduced glutathione (GSH), malondialdehyde (MDA), catalase (CAT), glutathione s-transferase (GST), and nitric oxide (NO) kits were purchased from Spectrum Diagnostic Company (Obour City, Egypt).

### Preparation of Clove (Syzygium aromaticum) Extract

Pieces of bud cloves were chopped, then dried. After that, the substance was ground into a fine powder, and 100 g of it was macerated in 1 L of 95% ethanol at room temperature for 48 h. After the solid sample was macerated three times, the liquid from the maceration was filtered using filter paper. 3.25 g of extracted product were obtained by gathering and drying the volume of the maceration using a rotary evaporator. The creation of NPs came after the outcomes of the clove bud ethanol extract.

### Preparation of Blank Chitosan-Alginate Nanoparticles (ChNPs)

The sodium alginate and calcium chloride solutions were prepared by dissolving the substances in distilled water. The pH of the sodium alginate solution was modified to 5.10 with hydrochloric acid. A specified quantity of chitosan was dissolved in a 1% acetic acid solution, and the pH was adjusted to 5.40 using NaOH. 2.00 ml of aqueous calcium chloride (3.35 mg/ml) were added dropwise to 10.00 ml of aqueous sodium alginate (3.00 mg/ml) while stirring for 30 min, after which 4.00 ml of chitosan solution (0.80 mg/ml) were incorporated into the resulting calcium alginate pre-gel and agitated for an additional hour. The resulting opalescent dispersion was equilibrated overnight to facilitate the formation of homogenous nanoparticle size [23].

### Synthesis of the Clove-Loaded Chitosan Nanoparticles (CNPs)

A clove solution was prepared by dissolving 2 mg of clove extract in a 2 ml alcohol/water combination (1:1). Subsequently, 300 ml of clove solution was integrated into the calcium chloride solution, after which the remaining procedures were identical to those employed in the creation of blank chitosan-alginate nanoparticles [24].

### Characterization of the (CNPs)

#### UV-Spectroscopy

UV-spectroscopy was employed to assess the optical absorption of CNPs in suspension (2 mg/ml). The experiment utilized a twin-beam UV–Vis spectrophotometer (Thermo Scientific, United States) at ambient temperature across a 200–800 nm wavelength spectrum.

#### Fourier Transform Infrared Spectroscopy (FTIR)

FTIR spectra were obtained using a Shimadzu 8400S spectrometer (SpectraLab Scientific, ON), with 128 scans in the 400 to 4000 cm-1 wave range.

#### Transmission Electron Microscopy (TEM)

Using a TEM (JEOL Inc., EM2100), the size of the CNPs was measured using an 80 kV accelerating voltage microscope and a 4-megapixel (2048 × 2048) bottom-mount CCD camera (AMT XR41-B).

#### Encapsulation Efficiency (EE%) and Loading Capacity (LC%)

0.25 g of CNPs was mixed with 5 mL of 2 M aqueous hydrochloric acid and refluxed at 95 °C for 30 min. Following cooling, 2 mL of 96% v/v ethanol was added to the mixture and centrifuged at 10,000 rpm for 5 min at 25 °C [25]. The clove content in the supernatant was analyzed by a UV–Vis spectrophotometer using UV absorbance at a wavelength of 280 nm. The amount of clove was calculated by calibration curve of pure clove in ethanol at 280 nm [26]. Encapsulation efficiency (EE%) and loading capacity (LC%) were estimated using the following equation. EE (%) = amount of encapsulated clove extract/initial amount of extract × 100. LC (%) = amount of encapsulated clove extract/amount of nanoparticles × 100 [27].

### Experimental Animals

In this study, 2 months old male Wistar albino rats (*Rattus norvegicus*) weighing 140 ± 10 g were used. Rats were received from the National Research Center (NRC, Dokki, Giza) and before the experiment began, the laboratory animals were given seven days to acclimate. Animals were housed in well-ventilated cages at 23–25°C, 47–54% relative humidity, and 12 h of dark/light cycle. Animals received a standard diet, chow, and water throughout the experiment.

### Acute Toxicity Study for the Synthesized Nanoparticles

The preliminary range of CNPs dose calculated using previous study [28]. Eighteen rats were divided into nine groups (2/group). The first group was considered the control, and the others were orally injected with various doses of ChNPs and CNPs, each separately (10, 100, 300, and 600 mg/kg). Following the administration, the animals were observed for an hour, followed by 10-min intervals every two hours for 24 h. Observing their deaths from the disease, the animals were examined for any changes in their behavior, such as paw licking, fatigue, semi-solid stools, salivation, writhing, and appetite reduction. The preliminary range-finding was determined using the formula X = (M0 + M1)/2 Where, X: Preliminary range-finding, M0: the highest dose of CNPs that caused no mortality, M1: the lowest dose of CNPs that caused mortality [28]. The no observed adverse effect level (NOAEL) at the highest dose that showed no mortality or severe symptoms was 45 mg/kg.

### Induction of the Obesity Model

For four weeks, the treated rats were fed a high-fat diet (HFD) in accordance with the methods described by Mohamed et al. [29]. 40% of calories from fat, 17% from protein, and 43% from carbs; this diet had an energy content of 5.3 kcal/g. The meal ingredients comprise 68% regular chow pellets, 20% milk powder, 6% ghee, and 6% maize oil (meal ingredients were purchased from the local market, Egypt and prepared fresh every day) [30].

### Experimental Design

After one week of acclimatization, 24 rats were divided into four groups (6 rats per group). Control group: rats were fed on regular chow pellets and oral daily distilled water administration for four weeks. HFD group: rats were fed on HFD and orally administered distilled water for four weeks. HFD + ChNPs: rats were fed on HFD along with ChNPs (45 mg/kg.b.wt) for four weeks. HFD + CNPs: rats were fed on HFD along with CNPs (45 mg/kg.b.wt) for four weeks. During the experiment, the animals were watched daily, and their weights were recorded weekly to track how many died and how much their weight changed.

### Sample Collection

At the end of the experiment, all rats were weighed and anesthetized with sodium pentobarbital 50.00 mg/kg [31]. The blood samples were collected by cardiac puncture. Both testes and both cauda epididymis were removed immediately after dissection, washed in saline, and weighed. The left testis of each animal was fixed in 10% neutral buffered formalin and prepared for histological examination. The right testis was frozen at − 20 °C for the physiological analysis. The left cauda epididymis from each rat was used for sperm count and abnormalities examination, and the right one was used for sperm motility examination. Of note, Biochemical and histological assessments were doing with Blinding in Assessments.

### Sample Preparation

#### Serum Preparation

The collected blood samples were centrifuged at 3000 rpm for 20 min. The collected serum was stored at −20° C until used for biochemical assays.

#### Testis Homogenate Preparation

Testis was homogenized (10% w/v) in ice-cold 0.10 M Tris–HCl buffers (PH 7.4). The homogenate was centrifuged at 3000 rpm for 5 min. At 4 ^°^C, the resultant supernatant was used for the physiological analysis.

### Sperm Analysis

The cauda epididymis was minced in 1.00 ml of 0.90% NaCl in an incubator at 37°C. The epididymal sperm concentration and motility were determined with a Neubauer hemocytometer. Sperm smears were made on clean slides, allowed to dry, and stained with Eosin and Hematoxylin stain. Approximately 300 sperm were analyzed on each slide. The overall abnormality rates of the sperm were noted and expressed as a percentage, and photomicrographs were taken to ascertain the percentage of morphologically abnormal sperm [32]. Fructose level in sperm samples was measured according to the method of Foreman et al. [33]. which indicates that a pink color is produced when fructose is heated with hydrochloric acid and resorcinol. This color can be directly measured using a spectrophotometer.

### Biochemical Assessment

Of note, Biochemical and histological assessments were doing with Blinding in Assessments.

#### Glucose, Total Protein, and Lipid Profile

Determination of Glucose, total protein, serum triglycerides (TG), serum total cholesterol (TC), low density lipoprotein cholesterol (LDL-C), and high density lipoprotein cholesterol (HDL-C) were done using kits purchased from Spectrum Diagnostic Company (Obour city, Egypt).

#### Fertility Hormones

Follicle-stimulating hormone (FSH), luteinizing hormone (LH), and testosterone (T) levels were determined according to the ELISA assay instructions.

### Determination of Testicular Oxidative Stress

In testis homogenates, malondialdehyde (MDA), nitric oxide (NO), glutathione reduced (GSH), catalase (CAT), and glutathione S-transferase (GST) were analyzed as oxidative stress biomarkers.

### Histological Examination

#### Haematoxylin and Eosin Staining

Following fixation in 10% neutral buffered formalin, testicular tissues were dehydrated in a series of ethyl alcohol (70%, 80%, 90%, 95%, and 100%), rinsed with xylene, embedded in paraffin wax, sectioned at 5–6 microns, and stained with haematoxylin and eosin (H&E) stain [34].

#### Picrosirus Red Staining

Tissue sections were dewaxed in xylene, then rinsed in absolute ethanol, and then three rinses in distilled water. Stain in Picrosirius red working solution (0.5 g of Direct Red 80 with 500 mL of 1.3% Picric Acid) for 1 h. Remove excess stain by washing twice with the wash solution (5 ml glacial acetic acid to 1 L of water). Dehydrate slides by immersing them in a series of increasing alcohol concentrations; transfer slides sequentially to dist. H_2_O, 70%, 80%, 90%, 95%, and 100% (v/v) ethanol solution. Finally, it was cleared in xylene and mounted with DPX.

### Analysis of Testicular Morphometry

Employing computer-aided microscopy and the "ImageJ" image analysis system, the average diameter and thickness of the germinative cell layer of seminiferous tubules were quantified in 10 randomly selected microscopic fields from each testicular section. Statistical methods were employed on the gathered data.

### Statistical Analysis

Data were presented as means ± standard error. SPSS, for Windows (version 25.0), was utilized to conduct the statistical analysis. A one-way analysis of variance (ANOVA) with Duncan post hoc test was employed to compare the group means, and statistical significance was determined at p < 0.05 to evaluate comparisons within groups.

## Results

### Characterization of the Clove-Chitosan Nanoparticles (CNPs)

#### Ultraviolet–Visible (UV–Vis) Spectroscopy

**Fig. 1:**
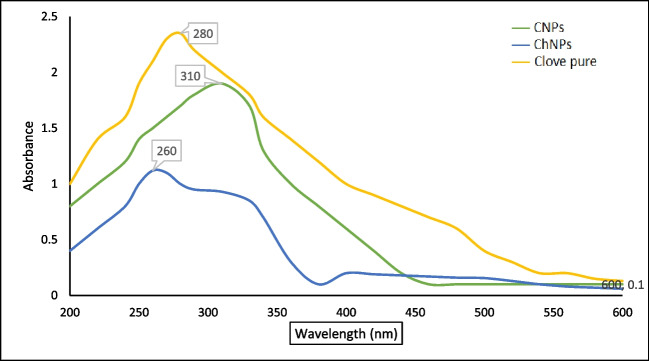
UV–visible absorption spectrum of the pure clove, ChNPs and CNPs

The optical properties of clove, ChNPs, and CNPs were investigated using UV–Vis. Spectroscopy. The absorption spectra of the pure clove were at 280 nm, ChNPs at 260 nm, and CNPs at 310 nm are shown in Fig. 1. The shift in clove peak of CNPs due to the interaction between the components and confirm encapsulation successfully.

#### Fourier-Transform Infrared Spectroscopy (FTIR)

**Fig. 2:**
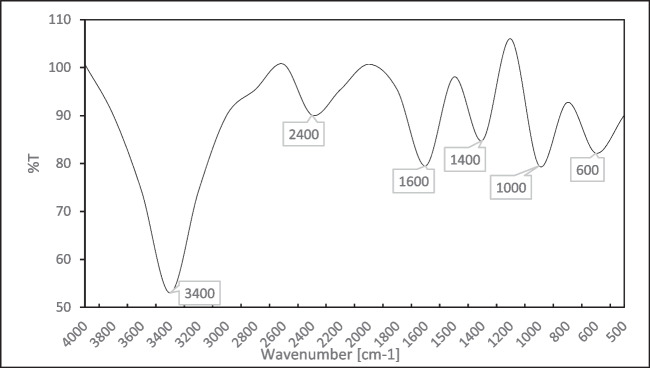
FTIR spectrum of the ChNPs

**Fig. 3:**
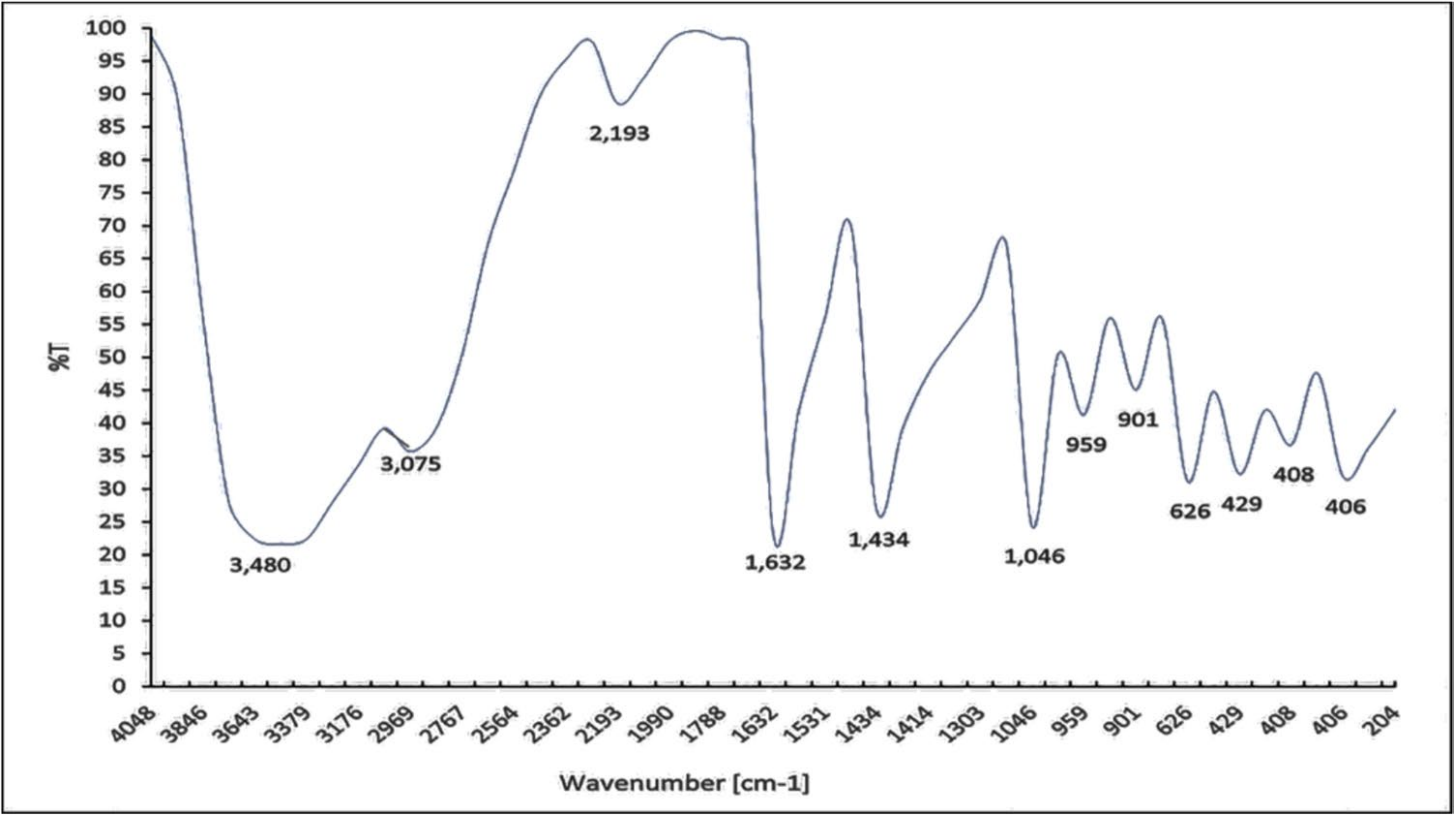
FTIR spectrum of the CNPs

To Look into the interactions between ChNPs and CNPs, FT-IR spectrum of ChNPs was analyzed (Fig. 2) showing characteristic peaks at almost 3400 cm^−1^ (OH and NH stretching), 2400 cm^−1^ (stretching vibration of CH_2_), 1600 cm^−1^ (amides), 1400 cm^−1^ (CN), 1000 cm^−1^ (C = O = C stretching). The FTIR analysis performed on CNPs. The OH group is represented by the very bright peak at 3489.68 cm^−1^, and the alkyl CH stretch (sp^3^) was detected at 3075.86 cm^−1^. A peak represented the C-O ester group at 2193.57 cm^−1^, and the 1632.80 cm^−1^ peak was characteristic of the aliphatic alkenes. The aromatic group is represented by a prominent peak at 1434.49 cm^−1^, while the presence of methylene (CH_2_) is indicated by a peak at 1046.27 cm^−1^. The presence of CH_2_ and C = C is indicated by weak bands at 959.54 and 901.38 cm^−1^ (Fig. 3).

### Transmission Electron Microscope (TEM) Analysis

**Fig. 4:**
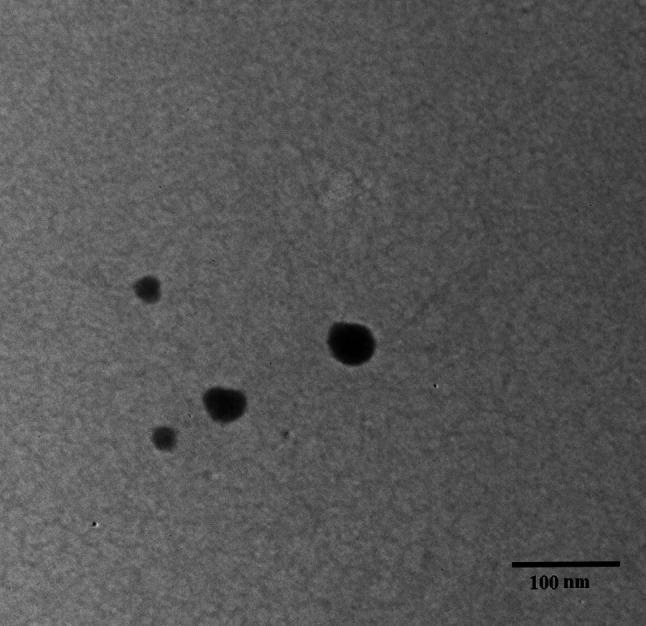
TEM micrograph of the CNPs

TEM analysis revealed the morphology of CNPs, which is depicted in Fig. 4. The CNPs exhibited a spherical structure with an average diameter of 22-25nm.

#### Encapsulation Efficiency (EE%) and Loading Capacity (LC %)

The LC% and EE% of CNPs were determined using a UV–VIS spectrophotometry test, and the LC% of CNPs was 11.50%, with an EE % was 81.84%.

### Body Weight and Reproductive Organs Relative Weights

A significant increase (p < 0.05) was reported in final body weight and weight gain in HFD rats; additionally, a significant decrease was noted in relative cauda weight (RCW) and relative testis weight (RTW) as compared to the control group. The administration of ChNPs and CNPs revealed a significant decline in final body weight and weight gain, as well as a significant increase in RCW and RTW compared to the HFD group (Table 1).

**Table 1:** Effect of ChNPs and CNPs on bodyweight and reproductive organ relative weights

	Control	HFD	ChNPs	CNPs
Final body wt. (g)	236.80 ± 2.50^a^	303.00 ± 6.10^c^	272.00 ± 7.90^b^	270.80 ± 3.70^b^
Weight gain (g)	27.40 ± 2.80^a^	94.20 ± 3.30^c^	42.00 ± 6.20^ab^	54.40 ± 12.80^b^
RCW (%)*	0.63 ± 0.00^c^	0.46 ± 0.03^a^	0.55 ± 0.02^b^	0.60 ± 0.03^bc^
RTW (%)^#^	0.45 ± 0.02^c^	0.35 ± 0.01^a^	0.41 ± 0.02^b^	0.42 ± 0.01^bc^

Values are given as means ± SEM (n = 6).

Each value not sharing a common letter superscript is significantly different (p < 0.05).

* Relative Cauda Weight (RCW) = cauda weight/final body weight × 100.

^#^ Relative Testes Weight (RTW) = testes weight/final body weight × 100.

**Fig. 5:**
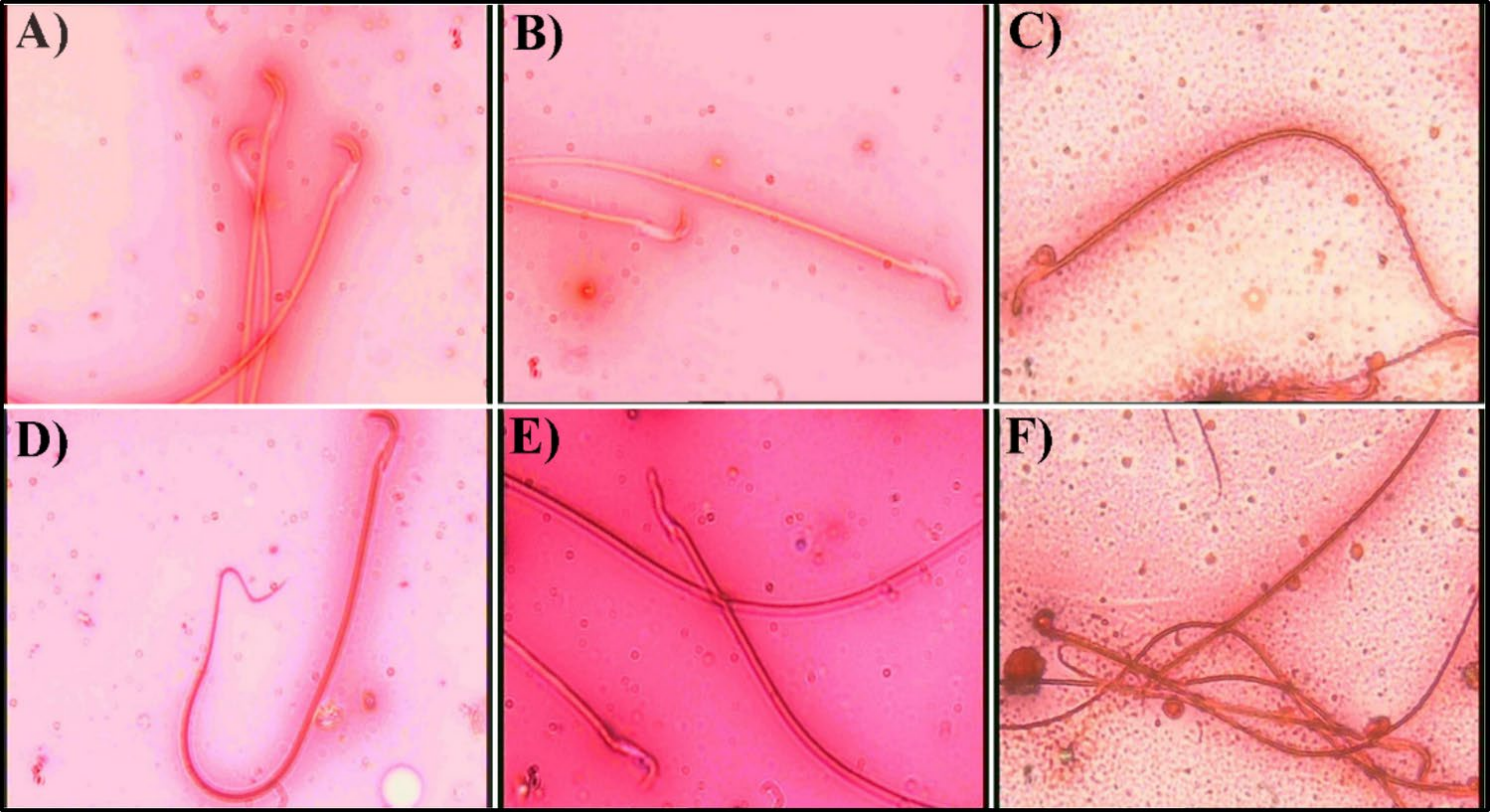
Photomicrographs of sperm morphology (H&E—× 400). (**A**) Sperm from a control rat with a normal shape, formed of a curved, hooked head and normal tail. Sperm deformed shapes from rats treated with HFD: (**B**) live banana-shaped head sperm. (**C**) dead banana-shaped head sperm. (**D**) dead sperm with a bent tail. (**E**) unhooked straight head sperm (**F**) amorphous head sperm

As presented in Table 2, a significant decrease was reported in sperm count, motility, morphological abnormality percentage, and fructose concentration, compared to control rats. The most common abnormal forms were amorphous head, unhooked head, and banana-shaped head of sperm (Fig. 5). Interestingly, rats that were orally administered ChNPs and CNPs showed a significant climb in sperm parameters. Moreover, CNPs have a greater effect than ChNPs in improving the sperm parameters.

**Table 2:** Effect of ChNPs and CNPs on the epididymal sperm parameters

	Control	HFD	ChNPs	CNPs
Count (million/ml)	103.67 ± 1.69^d^	53.67 ± 1.50^a^	72.50 ± 3.42^c^	91.00 ± 2.24^b^
Motility (%)	76.83 ± 3.13^c^	42.17 ± 2.77^a^	58.33 ± 2.17^b^	75.17 ± 1.30^c^
Abnormality (%)	11.33 ± 0.49^a^	27.50 ± 1.18^d^	21.50 ± 0.85^c^	18.50 ± 0.43^b^
Fructose (mmol/L)	30.91 ± 1.92^C^	13.82 ± 0.59^a^	23.58 ± 1.04^b^	27.51 ± 0.71^c^

Values are given as means ± SEM (n = 6).

Each value not sharing a common letter superscript is significantly different (p < 0.05).

### Biochemical Assessment

#### Glucose, Total Protein, and Lipid Profile

**Fig. 6:**
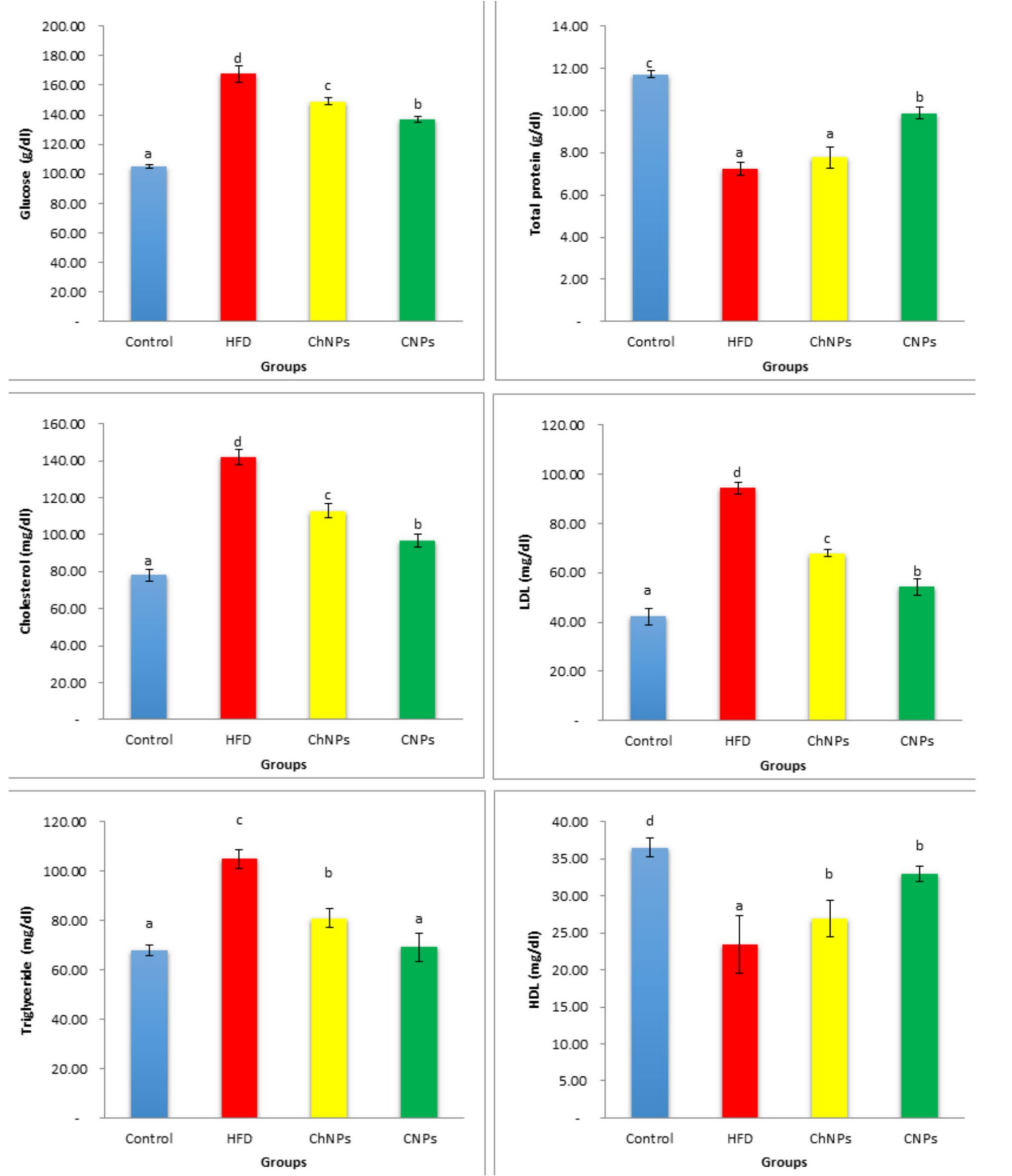
Histograms showing the effect of ChNPs and CNPs on glucose (g/dl), total protein (g/dl), cholesterol (mg/dl), Low-density lipoprotein (LDL) (mg/dl), high-density lipoprotein values (HDL) (mg/dl), and triglyceride levels (mg/dl) in HFD rats

The HFD group showed a significant increase in glucose, cholesterol, Low-density lipoprotein (LDL), and triglyceride levels but a reduction in total protein and high-density lipoprotein values (HDL) compared to the control group. In contrast to the HFD group, oral therapy of ChNPs and CNPs significantly retained the lipid profile values to normal levels. Furthermore, CNPs exert a more significant influence than ChNPs on the optimization of lipid profile (Fig. 6).

#### Fertility Hormones

Testosterone, FSH, and LH in HFD rats recorded a significant decrease compared to the control group rats. However, treated rats that were orally administered ChNPs and CNPs showed a significant increase in these fertility hormones compared to HFD rats. Meanwhile, CNPs induced a more remarkable increase in these aspects than ChNPs (Table 3).

**Table 3:** Serum testosterone, FSH, and LH levels

	Control	HFD	ChNPs	CNPs
Testosterone (ng/dL)	3.22 ± 0.06^d^	0.62 ± 0.03^a^	1.07 ± 0.06^b^	2.17 ± 0.05^c^
FSH (mIU/mL)	0.72 ± 0.03^d^	0.17 ± 0.02^a^	0.29 ± 0.03^b^	0.42 ± 0.03^c^
LH (mIU/mL)	0.56 ± 0.02^d^	0.14 ± 0.02^a^	0.24 ± 0.02^b^	0.34 ± 0.02^c^

Values are given as means ± SEM (n = 6).

Each value not sharing a common letter superscript is significantly different (p < 0.05).

As presented in Table 4, in HFD rats, a significant rise was reported in MDA, compared to the control group rats, and a significant decrease was reported in GSH, GST, NO, and CAT. Besides, ChNPs and CNPs rats showed a significant decrease in MDA and a significant increase in GSH, GST, NO, and CAT, compared to HFD rats. The improving effects of CNPs were superior to those of ChNPs in the oxidative stress parameters.

**Table 4:** Testicular oxidative stress parameters

	Control	HFD	ChNPs	CNPs
MDA (nmol/ml)	0.67 ± 0.07^a^	1.47 ± 0.07^d^	1.14 ± 0.07^c^	0.87 ± 0.03^b^
GSH (nmol/ml)	0.18 ± 0.01^d^	0.08 ± 0.00^a^	0.11 ± 0.00^b^	0.14 ± 0.01^c^
GST (U/g.tissue)	1.57 ± 0.08^C^	1.16 ± 0.04^a^	1.25 ± 0.01^ab^	1.40 ± 0.05^b^
NO (µmol/g.tissue)	114.72 ± 3.20^d^	31.97 ± 3.28^a^	72.61 ± 1.95^b^	87.79 ± 2.96^c^
CAT (U/g.tissue)	4.25 ± 0.39^C^	0.52 ± 0.06^a^	0.95 ± 0.05^ab^	1.37 ± 0.08^b^

Values are given as means ± SEM (n = 6).

Each value not sharing a common letter superscript is significantly different (p < 0.05).

### Histological Examination of the Testicular Tissue

#### Haematoxylin and Eosin Staining

**Fig. 7:**
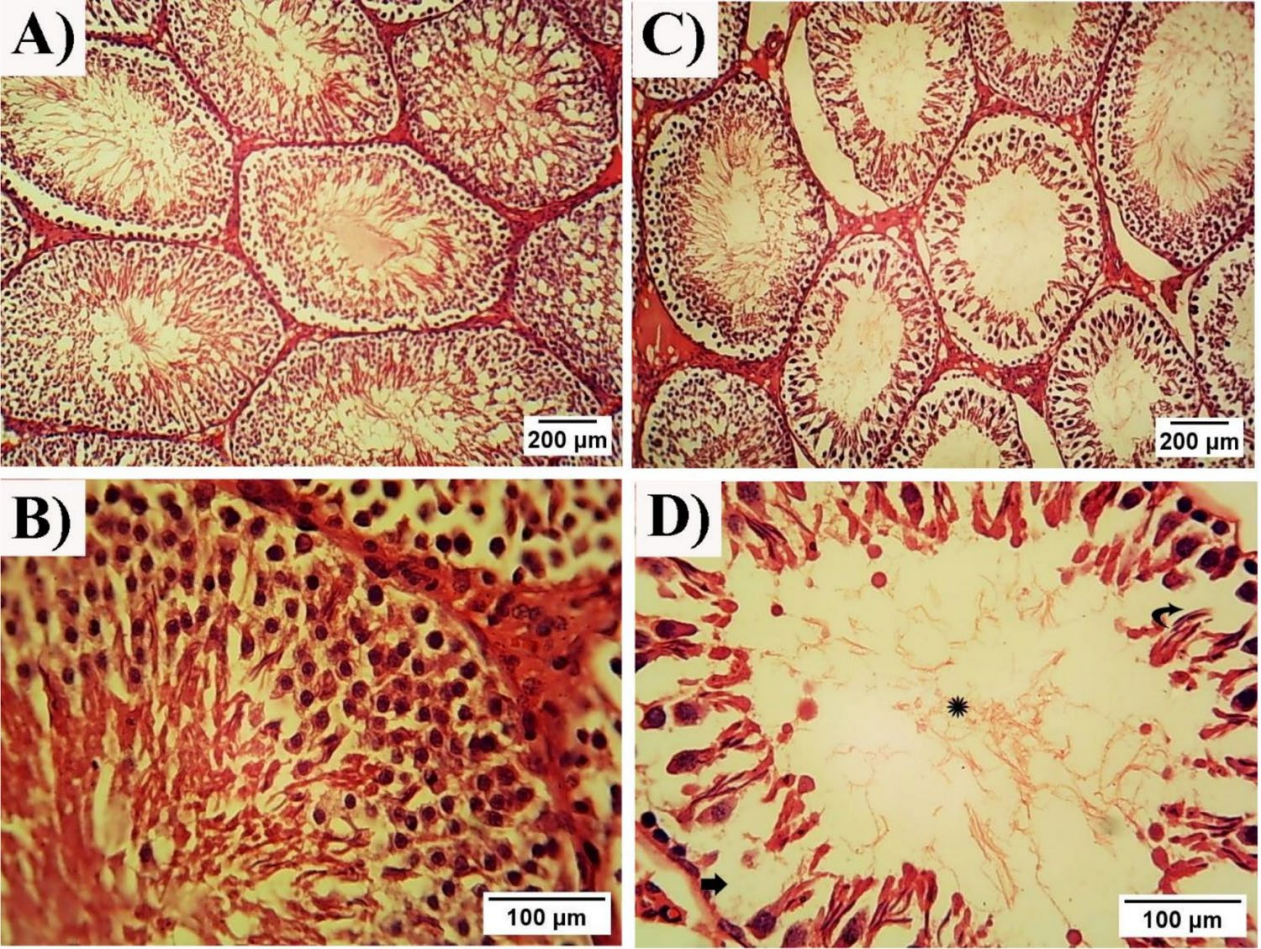
Photomicrographs of a cross-sections of the testes (hematoxylin and eosin). **A&B**: Control group showing normal structure of the testicular tissue with nearly equal size and shape of the seminiferous tubules and normal germinal epithelium with stratified spermatogenic cells, intact basement membrane, and normal interstitial tissue. **C&D**: HFD group showing depletion of the seminiferous epithelium, destruction of the interstitial tissue, tubular lumen with few sperms (star), decrease in the spermatogenic cells (thick arrow), and reduction in the thickness of seminiferous epithelium (curved arrow) (400x)

**Fig. 8:**
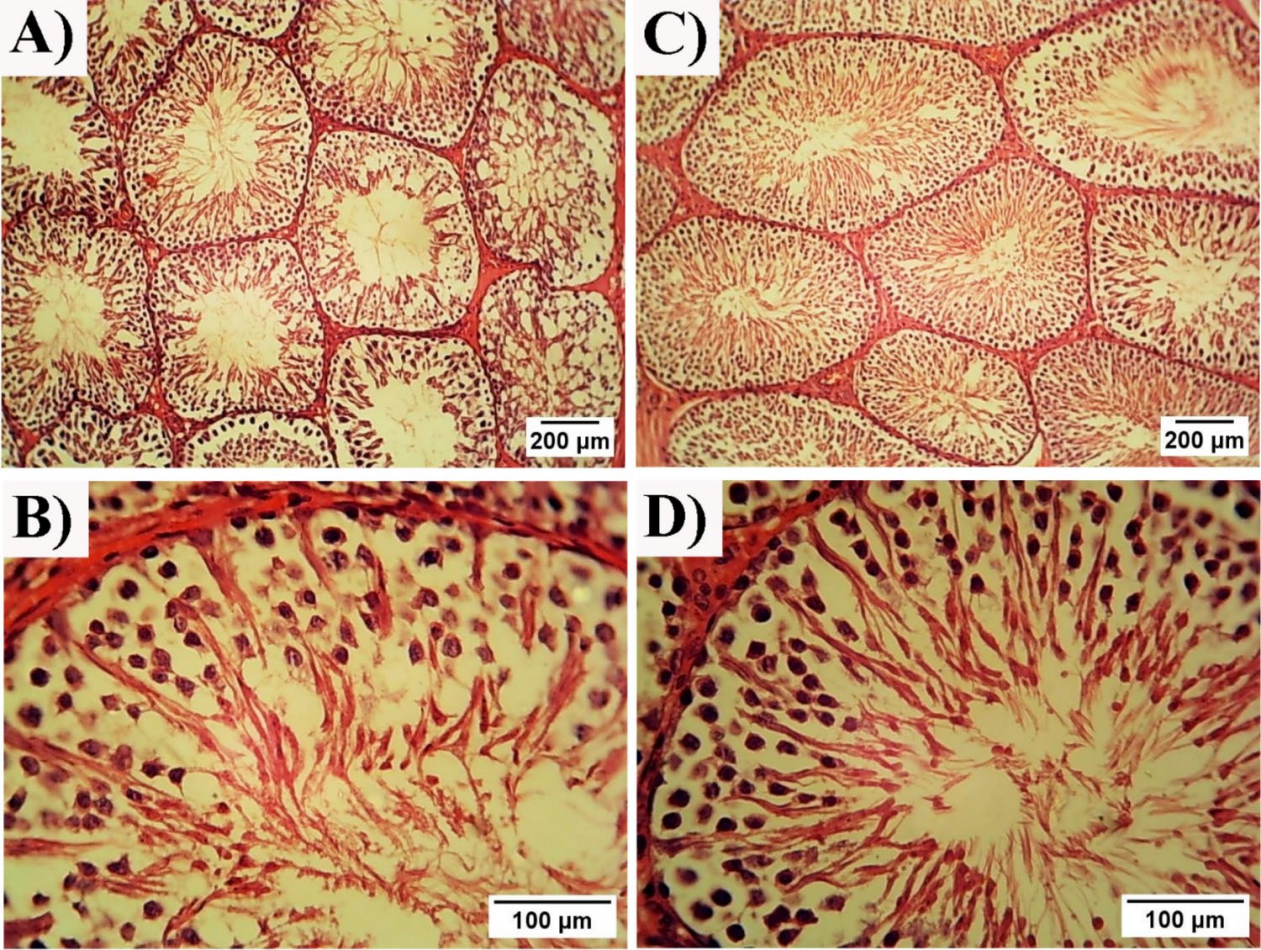
Photomicrographs of a cross-sections of the testes (hematoxylin and eosin). **A&B**: ChNPs group showing quiet improvement and restoration of structure and thickness of the testicular tissue in some tubules. **C&D**: CNPs group showing restoration of the typical structure of the testicular tissue with intact germinal epithelium

The testes of the control group showed nearly equal-sized and shaped seminiferous tubules with normal spermatogenic cells, Sertoli cells, interstitial tissue, typical characteristics of seminiferous epithelium, and large diameters and intact basement membranes. Each tubule was lined by many layers of spermatogenic cells with obvious Sertoli cells in between. Sperms were seen in the lumen of the tubules. Seminiferous tubules were bound by basal laminae and myoid cells. The interstitial spaces between the tubules were occupied by the connective tissue, Leydig cells, and were accompanied by blood vessels (Fig. 7 A&B). The Testicular section of the HFD group showed atrophic seminiferous tubules with smaller diameters. The testicular damage was observed in almost all seminiferous tubules, including vacuolation, reduction in the thickness of seminiferous epithelium, and detached germinal epithelium from the basement membrane. Depletion of the seminiferous epithelium and destruction of the interstitial tissue were also observed. Some tubular sections of the HFD-treated rats showed disorganized seminiferous epithelium with discontinuous germ cell layers without sperms in their lumen. There was a large decrease in the number of germ cells and Sertoli cells with empty spaces in between. Many cells revealed cytoplasmic vacuolation; some exhibited deeply stained nuclei and separation of the germ cells from the basal laminae (Fig. 7 C&D). In the ChNPs group, the testicular section showed quite an improvement compared to the HFD group, hypo spermatogenesis, low number of sperms are found, and some degeneration in the germinal epithelium (Fig. 8 A&B). The histological examination of the testes of the CNPs group showed good improvement, good spermatogenesis, most of the tubules appeared to be filled with spermatogenic cells, intact spermatogonia were observed with restoration of the normal structure, and the thickness of the germinal layer. However, some seminiferous tubules showed occasional vacuolated cells with deeply stained nuclei. Normal clusters of Leydig cells occupied the interstitial spaces between the tubules with minimal empty spaces (Fig. 8 C&D).

#### Picrosirus Red Staining

**Fig. 9:**
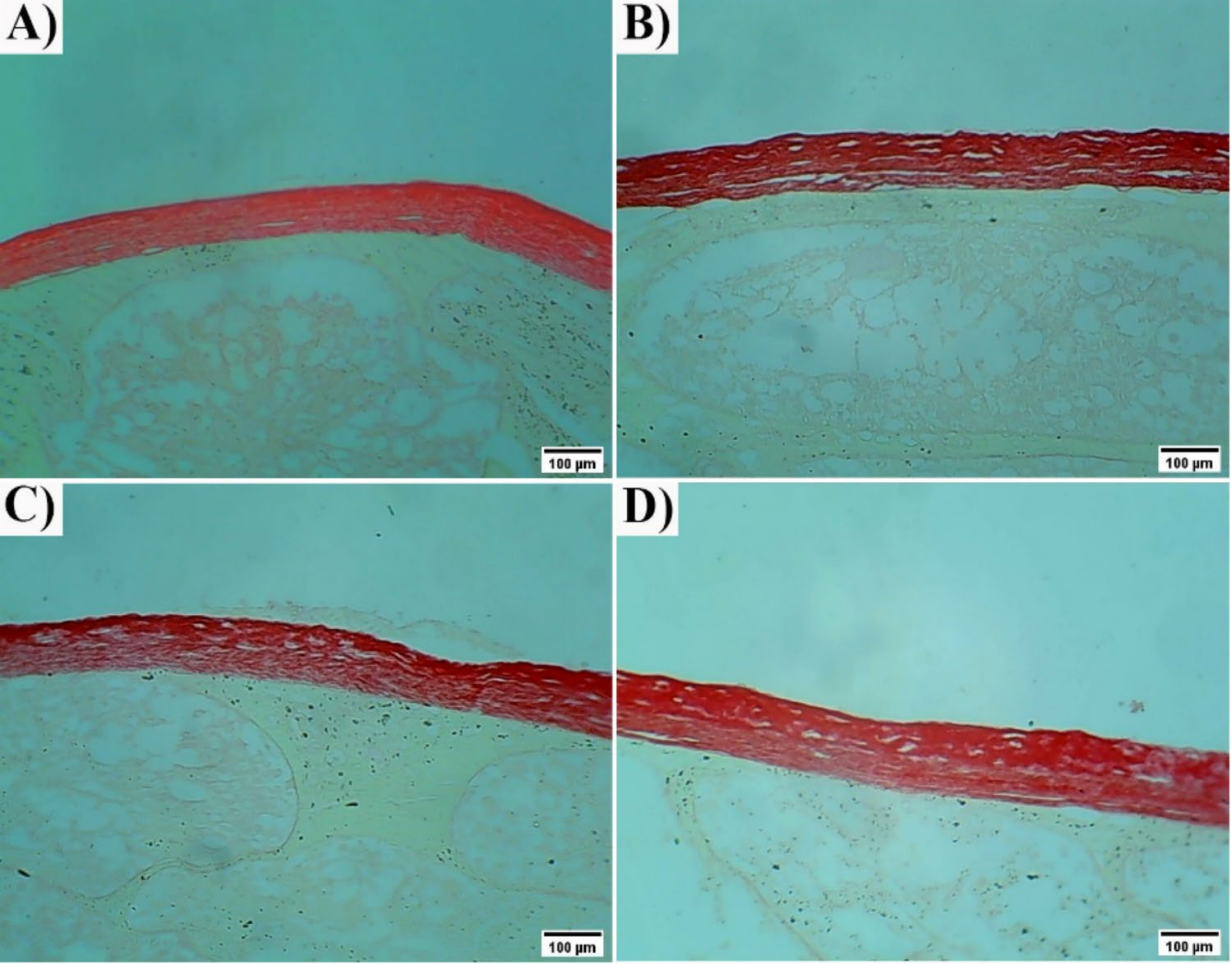
Photomicrographs of a cross-sections of the testes (Picrosirius red stain). **(A):** Control group showing intact tunica albuginea. **(B):** HFD group. **(C):** ChNPs group. **(D):** CNPs group

The assessment of Picrosirius red-stained sections revealed intact tunica albuginea with few collagen fibers in the control group (Fig. 9 A). While in the HFD-treated group, many collagen fibers in the tunica albuginea and the interstitial tissue were detected, with degeneration noticed in the tunica albuginea (Fig. 9 B). ChNPs and CNPs rats showed few collagen fibers in the tunica albuginea (Fig. 9 C&D).

### Analysis of Testicular Morphometry

The thickness of the germinal epithelium and the diameter of testicular tubules in HFD rats decreased significantly compared to the control group. However, the thickness increased significantly and the diameter increased non-significantly (P > 0.05) in the ChNPs group and CNPs group, as compared to the HFD group (Table 5).

**Table 5:** Testicular morphometric measurements

	Control	HFD	ChNPs	CNPs
Thickness of the germinal epithelium (µm)	87.10 ± 3.64^d^	33.20 ± 1.27^a^	51.30 ± 1.27^b^	71.30 ± 2.21^c^
Diameter of testicular tubules (µm)	268.20 ± 11.60^b^	230.90 ± 5.90^a^	232.10 ± 4.80^a^	247.80 ± 6.20^a^

Values are given as means ± SEM (n = 6).

Each value not sharing a common letter superscript is significantly different (p < 0.05).

## Discussion

The results of the present study indicated that the final body weights were significantly increased in the HFD group. Similarly, several studies disclosed the present findings [35–37]. However, the relative cauda weight (RCW) and relative testis weight (RTW) were significantly decreased in the HFD group [38, 39]. One of the main contributors to the development of obesity is high-fat consumption. Caloric surplus High-fat diets are energy-dense, providing significantly more calories per gram than standard or low-fat diets. The hyper-caloric nature of HFDs increases total caloric intake, leading to a positive energy balance and weight gain [40]. Alterations in energy expenditure, such as feeding rats a high-fat diet, have been shown to reduce energy expenditure by impairing thermogenesis and physical activity levels. This is partly due to diminished mitochondrial function in brown adipose tissue, which is critical for energy dissipation. Consequently, reduced energy expenditure contributes to fat accumulation and weight gain [41]. High-fat diets (HFDs) have been linked to numerous physiological and metabolic disruptions in male rats, including the reduced testis weight [42]. This phenomenon can be attributed to hormonal imbalances, oxidative stress, inflammation, and direct effects of lipid accumulation in reproductive tissues. Below is an analysis of potential mechanisms behind the reduction in testis weight observed in male rats fed HFDs, supported by research findings [43]. The treatment with Clove NPs caused a significant decrease in the final body weight of HFD rats in accordance with the results of the previous studies [44, 45]. This improvement in body weight gain may be associated with improving lipid profile, liver function, glucose level, antioxidant system, and reduction of the severity of fatty liver disease, which collectively lead to better metabolism and growth [46]. In addition, chitosan supplementation is thought to influence body weight and BMI by mechanisms that decrease fat absorption rates [47].

Regarding the serum total proteins, there was a significant decrease in the HFD groups. In agreement, many studies disclosed this findings [48, 49]. Evidence suggests that obesity can greatly impact total protein and eventually affect reproductive capacity. Total protein levels decrease in high-fat diet-related diseases primarily due to liver dysfunction, chronic inflammation, kidney damage, impaired amino acid metabolism, and gut dysbiosis. These factors collectively impair protein synthesis and increase protein degradation or loss. Obesity is commonly associated with liver damage resulting from cellular apoptosis and It is likely to be the primary mechanism by which HFD induces a decrease in total protein [50]. Hepatocyte injury in rats subjected to a high-fat diet is attributable to oxidative damage in tissues [35]. Monitoring total protein and albumin levels in individuals with HFD-induced metabolic disorders can provide insights into their nutritional and metabolic status [51]. Clove NPs administration caused a significant increase in the total protein of HFD rats. In accordance with the present results, Kumar et al.[52] recorded a significant increase in total protein in HFD-induced liver damage rats after oral administration of clove extracts. Clove extract contains phenolic compounds, including eugenol, which demonstrate beneficial hepatoprotective properties and may prevent chemically induced dyslipidemia, inflammatory responses, and mitochondrial oxidative damage in rat hepatocytes, hence protecting the liver's protein synthesis capability [53]. Furthermore, the hepatoprotective effects of chitosan may potentially be contingent upon its anti-inflammatory qualities and antioxidative activity [54].

Many studies have shown that glucose significantly increased in HFD groups, similar to our findings [55–57]. A high-fat diet (HFD) is strongly linked to metabolic disorders such as insulin resistance. Excess fat intake accumulates free fatty acids (FFAs) and toxic lipid metabolites (ceramides and diacylglycerols) in muscle and liver tissues. These lipids disrupt the insulin signaling pathway, particularly AKT phosphorylation, impairing glucose uptake [58]. High-fat diet-induced disease leads to glucose accumulation in the blood due to insulin resistance, increased glucose production, inflammation, oxidative stress, gut dysbiosis, and pancreatic dysfunction. These mechanisms collectively disrupt glucose metabolism, resulting in persistent hyperglycemia and a higher risk of metabolic disorders like type 2 diabetes [59]. The treatment with Clove NPs caused a significant decrease in the glucose levels of HFD rats. Similarly, Al-Trad et al. [60] recorded a significant decrease in glucose in HFD-induced hyperglycemia and insulin resistance in rats after an oral mixed diet of clove. The reduction in glycemic levels by eugenol has been evidenced through the inhibition of carbohydrate-metabolizing enzymes and the potential suppression of hepatic glucose synthesis and glucose uptake by skeletal muscle cells [61]. Furthermore, the antidiabetic properties of chitosan encompass glucose reduction, lipid reduction, antioxidative effects, anti-inflammatory effects, and gastrointestinal regulation [62].

With respect to the lipid profile, cholesterol, low-density lipoprotein (LDL), and Triglycerides significantly increased in HFD groups, while the High-density lipoprotein (HDL) significantly decreased. Previous studies confirmed the present findings [63, 64]. A high-fat diet raises cholesterol levels through several mechanisms, including increased dietary absorption, enhanced production in the liver, reduced cholesterol clearance, greater lipoprotein synthesis, inflammation, and gut microbiota alterations. While certain fats (saturated and trans fats) have a more detrimental effect, healthier fat choices (monounsaturated and polyunsaturated) can help regulate cholesterol levels and reduce cardiovascular risks [65]. HDL levels decrease in HFD due to hepatic lipid accumulation, impaired cholesterol transport, systemic inflammation, increased triglyceride levels, and oxidative stress[66]. Also, an increase in triglycerides due to excessive dietary fat intake, increased liver production of triglycerides, insulin resistance, reduced fat breakdown, and impaired clearance from the blood. Triglyceride levels require a balanced diet, emphasizing healthy fats and limiting saturated and trans fats [67]. The treatment with CNPs enhances the lipid profile, indicating their hypolipidemic effect. Harb et al. [46] reported that eugenol reduces cholesterol and hepatic steatosis in hypercholesterolemic rats by modulating the TRPV1 receptor. In addition, Xu et al. [68] indicated that chitosan improves lipid metabolism by regulating total cholesterol and LDL-C levels via the elevation of hepatic LDL receptor mRNA expression, hence facilitating the excretion of fecal bile acids.

The results of the present study indicated that sperm count, sperm motility, semen fructose, FSH, LH, and testosterone levels were significantly decreased in HFD groups. Evidence suggests that obesity can greatly impact sperm parameters, resulting in a decrease in testicular volume, a decline in sperm quality, and impaired spermatogenesis, eventually affecting reproductive capacity [69]. Moreover, HFD produces high levels of reactive oxygen species (ROS), which can destroy nearly all macromolecules, including DNA, protein, lipid, and carbohydrates. ROS have been linked to reduced testosterone levels, sperm motility, and sperm count [70]. Also, Obesity is associated with adipocyte hypertrophy and hyperplasia, which lead to changes in endocrine regulation in men, primarily through the secretion of adipokines. These physiological changes detrimentally impact the male reproductive endocrine system, primarily via the Hypothalamic–Pituitary–Gonadal (HPG) axis [71]. Moreover, Changes in the testicular tissues of male rats fed HFD, as observed under hematoxylin and eosin staining, are linked to multiple physiological disruptions. HFD can lead to decreased steroidogenesis, impaired spermatogenesis, increased apoptosis of germ cells, and compromised blood-testis barrier integrity. These effects are often mediated by oxidative stress, hormonal imbalances (e.g., reduced testosterone), and mitochondrial dysfunction. Besides, Sertoli cells, like "nurse" cells, support germ cell development structurally and metabolically. Lack of support may harm germ cells and hinder spermatogenesis [72]. The vacuolation and disintegration of the seminiferous epithelium in high-fat diet rats result from multiple reasons, including compromised blood-testis barrier integrity, oxidative stress, endocrine disturbance, and aberrant lipid buildup. Additionally, Vacuolization displaces seminiferous epithelium [73]. Moreover, molecular pathways like SIRT1/NRF2/MAPKs are disrupted, contributing to testicular tissue damage and fertility decline [74].

The CNP group showed an improvement in semen profile and reproductive hormones. Also, the treatment with CNP shows that normal seminiferous tubules, spermatozoa containing lumen, and interstitial cells of Leydig caused an improvement in the testes of rats. The positive impact of CNPs on fertility parameters is due to the antioxidant properties of clove or its direct influence on the hypothalamic–pituitary–gonadal axis, or both [75].

Oxidative stress facilitates tissue damage and cellular apoptosis, exhibiting a detrimental effect in conditions such as inflammation, aging, cardiovascular and neurological diseases, autoimmune disorders, cancer, and reproductive system changes[76]. Oxidative stress significantly contributes to sperm cell dysfunction. It is a primary factor in the etiology of male infertility due to the compromise of both the structural and functional integrity of spermatozoa [77]. Malondialdehyde (MDA), primarily a reactive aldehyde resulting from lipid peroxidation, has gained prominence as a valuable biomarker for gauging oxidative stress levels within biological samples [78]. Nitric oxide (NO) is a free radical recognized to play a regulatory role in the process of spermatogenesis at low concentrations, but at elevated levels, it leads to the formation of nitrogen-based reactive oxygen species (ROS), which are detrimental to the testicular tissue [79]. Catalase is a vital antioxidant enzyme that can balance the redox system by regulating antioxidant defenses against oxidative stress [80]. Glutathione S-transferases (GSTs) are members of the Phase-II detoxification enzyme family, which protects cellular macromolecules against reactive electrophiles by interacting electrophilic and hydrophilic compounds with glutathione [81]. Glutathione (GSH) is the main antioxidant in mammals, which is closely related to reproductive function. Simultaneously, decreased sperm quality is associated with decreased glutathione content and trace element deficiency in semen, which may be an important indirect biomarker of idiopathic male infertility. In addition, glutathione deficiency can lead to instability of sperm midsection and sperm motility defects [82]. The present study indicated that the GST, GSH, and CAT were significantly decreased in the HFD group. Generation of ROS is a common feature of cells, including mammalian sperm. Sperm are mitochondria-containing cells, which are a major source of ROS due to electron leakage, triggered by various factors that disrupt the electron transport chain and the NADH-dependent oxidoreductases. It is acknowledged, however, that physiological levels of ROS mediate essential sperm functions[83].

The current study revealed that the treatment with Clove NPs with its specific antioxidant properties protected the testis from oxidative stress produced by HFD. Previous investigations [84, 85]. have demonstrated that clove may serve as an antioxidant, reducing protein glycation and lipid peroxidation. The primary component of clove oil is eugenol, which is credited with numerous antioxidant effects through mechanisms including radical scavenging and metal ion chelation [21]. Furthermore, the hydroxyl and amino groups in chitosan are the principal functional groups responsible for its antioxidant action [86].

## Conclusion

CNPs alleviate testicular dysfunction caused by HFD by reducing hyperlipidemia and hyperglycemia, restoring sex hormone levels, enhancing spermatogenesis, and balancing oxidants-antioxidants system. CNPs showed a leading effect on fertility compared to chNPs used independently.

## Data Availability

The data supporting this study's findings are available from the corresponding author upon request.
